# Supplementary material for: Wild or Reared? Cassiopea andromeda Jellyfish as a Potential Biofactory
Source: Mar Drugs. 2025 Jan 1;23(1):19. doi: 10.3390/md23010019 (PMC11767235; doi:10.3390/md23010019)

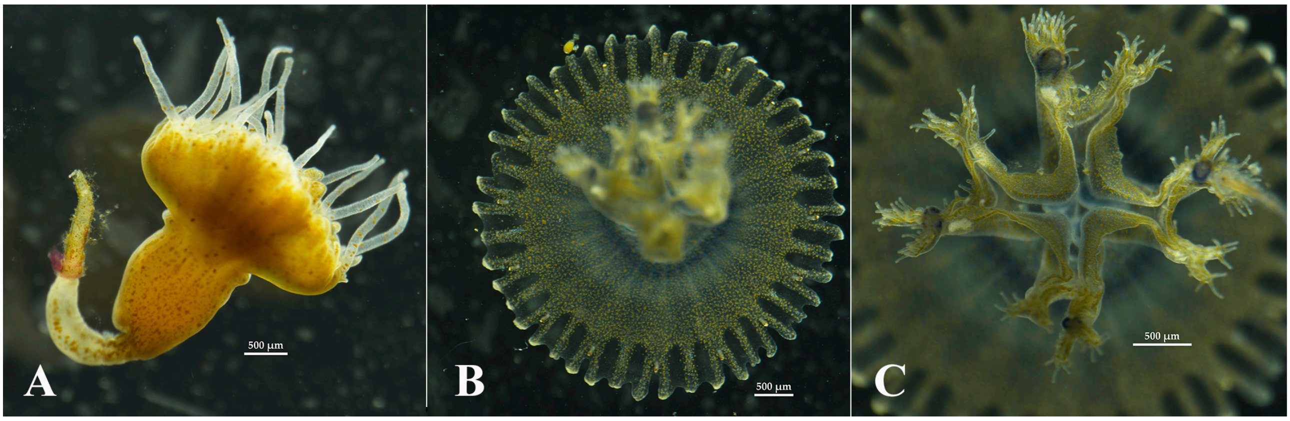

Figure S1 Light microscope images of *C. andromeda* polyp (**A**) and ephyrae (**B, C**) reared in aquarium under controlled conditions, ephyrae were born in aquarium. The zooxanthellae are visible as brown spots in all the tissues of polyp (**A**) and in umbrella (**B**) and oral arms (**C**) of ephyrae. Bar: 500 µm.

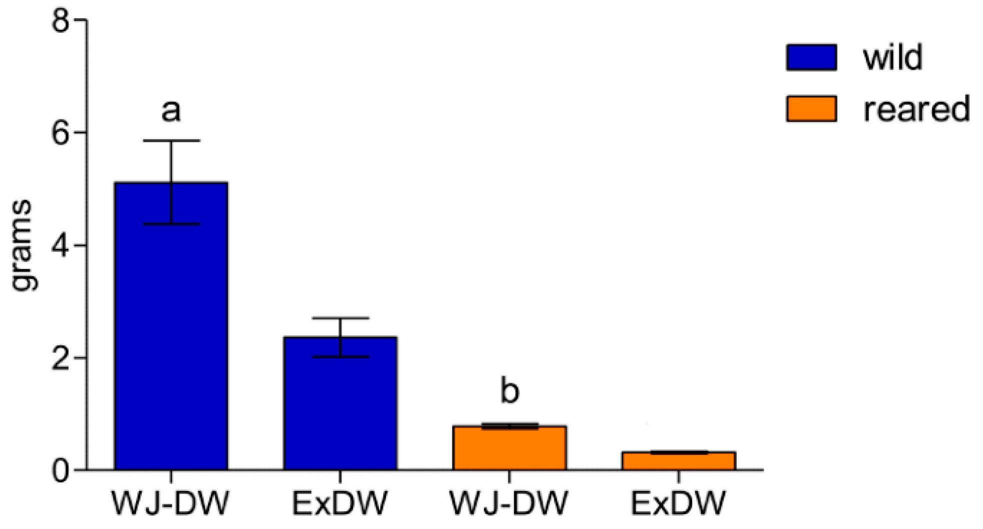

Figure S2. Dry weight of the whole jellyfish biomass (WJ-DW) and 80% ethanol extract (ExDW) obtained from each wild (blue) and reared (orange) jellyfish *C. andromeda* specimen. Values are expressed in grams and are means of freeze-dried biomass values of ten wild and reared *C. andromeda* specimens. Data are analyzed by unpaired t-test two-tail P value (α= 0.05).

Table S1. Fatty acids (FA) composition in wild *C. andromeda* jellyfish (WJ) as reported in [15, 24].

**Fatty acid (FA)**	**Whole Jellyfish** (WJ)	**80% EtOH Extract (ExDW)**	**Upper Phase (UP)**	**Lower Phase (LP)**
Saturated FA (SFA) %				
Lauric acid *C12:0*	9.3 ± 0.9	1.5 ± 0.2	8.5 ± 0.8	-
Myristic acid *C14:0*	4.2 ± 0.4	5.2 ± 0.5	2.5 ± 0.3	-
Pentadecanoic acid *C15:0*	-	-	-	-
Palmitic acid *C16:0*	21.9 ± 2.2	13.9 ± 1.4	25.2 ± 2.5	-
Margaric acid *C17:0*	-	-	-	-
Stearic acid *C18:0*	12.5 ± 1.2	9.9 ± 0.9	3.6 ± 0.4	-
Arachidic acid *C20:0*	0.6 ± 0.1	0.6 ± 0.1	-	-
**Total SFA**	**48.5 ± 4.8**	**31.1 ± 3.1**	**39.8 ± 4.0**	**-**
Monounsaturated FA (MUFA) %				
Palmitoleic acid *C16:1* (ω7)	4.3 ± 0.4	3.3 ± 0.3	2.8 ± 0.3	-
Margaroleic acid *C17:1*	-	-	-	-
Oleic acid *C18:1 cis-9*	2.8 ± 0.3	2.3 ± 0.3	-	-
Isoleic acid *C18:1 trans-10*	0.5 ± 0.1	0.6 ± 0.1	3.0 ± 0.3	-
Vaccenic acid *C18:1 trans-11*	-	-	-	-
Gondonic acid *C20:1 cis-11*	-	-	-	-
Paullic acid *C20:1 cis-13*	-	-	-	-
**Total MUFA**	**7.5 ± 0.8**	**6.1 ± 0.6**	**5.8 ± 0.8**	**-**
Polyunsaturated FA (PUFA) %				
Linoleic acid *C18:2 cis-9,12* (ω6)	0.8 ± 0.1	1.9 ± 0.2	-	-
Isolinoleic acid *C18:2 cis-6,9* (ω9)	0.5 ± 0.1	-	-	-
α-Linolenic acid *C18:3 cis-9,12,15* (ω3)	2.6 ± 0.3	3.2 ± 0.3	2.4 ± 0.3	-
γ-Linolenic acid *C18:3 cis-6,9,12* (ω6)	-	-	-	-
Stearidonic acid *C18:4* (ω3)	7.4 ± 0.7	7.9 ± 0.8	16.3 ± 1.6	-
Eicosadienoic acid *C20:2* (ω6)	-	-	2.7 ± 0.3	-
Dihomo-γ-linolenic acid *C20:3* (ω6)	-	-	-	-
Arachidonic acid *C20:4* (ω6)	14.2 ± 1.4	19.2 ± 1.9	14.4 ± 1.5	-
Eicosapentaenoic acid *C20:5* (ω3)	2.1 ± 0.2	3.5 ± 0.3	2.4 ± 0.3	-
Docosatetraenoic acid *C22:4* (ω6)	2.9 ± 0.3	4.2 ± 0.4	-	-
Docosapentaenoic acid *C22:5* (ω6)	2.5 ± 0.2	5.1 ± 0.5	2.2 ± 0.2	-
Docosahexaenoic acid *C22:6* (ω3)	11.0 ± 0.1	17.8 ± 1.8	14.0 ± 1.4	-
**Total PUFA**	**44.0 ± 4.4**	**62.8 ± 6.3**	**54.4 ± 5.5**	**-**
**Total fatty acids (%)**	**100.0**	**100.0**	**100.0**	**0**
Σω6	*17.9*	*25.3*	*17.2*	
Σω3	*25.6*	*37.5*	*37.3*	
**Ratio *ω6/ω3***	***0.7***	***0.7***	***0.5***	

Figure S3: Chromatographic profile of HPLC analysis of the lipidic fraction (Upper Phases) of the 80%ethanol extracts (ExDW) of reared jellyfish *C. andromeda*. Absorbance was recorded at 450 nm for peridinin, diadinoxanthin, and lutein [15]. Compounds were confirmed by comparison with the retention time and absorbance spectra of pure standard compounds. Peaks: 1 = peridinin isomer 1; 2 = peridinin isomer 2; 3 = peridinin isomer 3; 4 = diadinoxanthin; 5 = lutein.

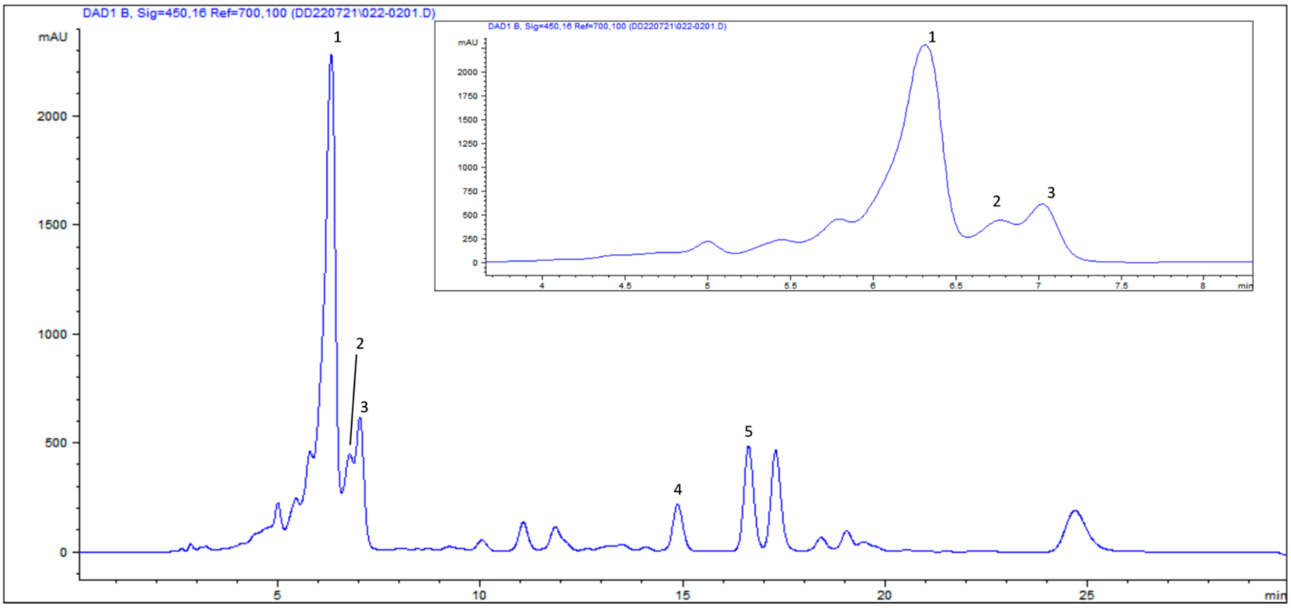

Supplement: Supplementary file 1 [file marinedrugs-23-00019-s001.zip › marinedrugs-3352196-Supplementary.pdf]
